# Some Unusual Cases

**Published:** 1909-07

**Authors:** A. W. Fly

**Affiliations:** Galveston, Texas


For Texas Medical Journal.

### SOME UNUSUAL CASES.

BY A. W. FLY, M. D., GALVESTON, TEXAS.

Case No. 1.—In the early part of March, 1908, I was called suddenly to see Mr. T. B. N., age 34, who was complaining of severe pain in the abdominal region, with great distension. Being a heavy feeder and a fair boozer, I was under the impression he was suffering from a case of acute auto-intoxication; gave him a hypodermic of a twelfth of a grain of heroin to quiet him for the night; saw him about daylight the next morning. The abdominal distension had increased; the pain was more acute. After a careful examination I diagnosed the case as fulminating appendicitis. As soon as I could get him to the infirmary and make the necessary preparation, after an administration of anesthetic, I made an incision about three inches long over the region of the appendix, the object of which was to promote free drainage. I found the abdominal cavity distended with thick, tenacious pus. The appendix was gangrened and disconnected from the bowel. All I had to do was to remove it with a pair of forceps. After thoroughly mopping out the abdominal cavity I packed the wound from the bottom with iodoform gauze 5 per cent, dressed the wound three times a day, irrigated it with peroxide of hydrogen one to two thousand solution of bichloride of mercury; confined him to liquid food exclusively, and each morning had administered two teaspoonfuls of Rochelle salts in a glass of water. The third morning I had an enema given and the water came through the external wound, showing that a fistula had resulted. I then placed two drainage tubes, one above and the other below the fistula, so as to relieve the pressure at the point of fistula, and packed with gauze on either side and between the tubes; continued to change the dressing three times a day until the fistula entirely healed; then dressed the external wound twice a day. At the expiration of two months the external wound was entirely healed and he has been perfectly well to the present time.

Case No. 2.—About the first of August, 1908, I was called to see a lady patient, the mother of one child about three years old, who was complaining of some pain in the region of the appendix, but as she was in a state of pregnancy I did not make as thorough an examination as I otherwise would; but about ten days later she had another attack, suffering somewhat more than the first. I continued to temporize, hoping that I might be able to carry her through without any radical measures. On the night of the 20th of August, about 11 o'clock, I was called to see her and found her suffering violent pain in the region of the appendix, extreme nausea, the right limb drawn up, and high temperature. I saw the condition was serious, and, notwithstanding she was four months pregnant it was either a question of doing a radical operation at that time and saving both mother and child, or losing both. In the absence of the husband I submitted my opinion to the mother, who told me to take the responsibility and do the best I could. On the morning of the 22d of August I made a free incision over the region of the appendix, found it highly inflamed and bound down to the apex; removed that, and on further examination found the tube and ovary on that side were badly diseased, the tube inflamed and bound down to the surrounding structure.

The ovary was badly diseased and studded with cyst. I then removed the ovary and tube on that side. She progressed to a full recovery, and on the 18th of January, 1909, I delivered her of an eight-pound healthy girl baby. Mother and child are both sound and healthy. The mother says she feels better now than she did during her girlhood.

Case No. 3.—About April 1, 1909, I was called to see a lady, eight and a half months advanced in pregnancy, who was having a slight hemorrhage. I tamponed the vagina with 5 per cent iodoform gauze, with instructions to the nurse to report to me should any further hemorrhage occur. I saw her early next morning and found the womb somewhat dilated, and upon examination found she had placenta previae. When I removed the gauze there was quite a gush of blood. I then used artificial dilatation and plugged the uterus with gauze, with instructions to prepare for artificial delivery at 11 o'clock. At 11 o'clock I promptly had her placed under an anesthetic, completed dilatation, inserted my hand and arm into the uterus and brought down the feet and delivered without any unusual difficulty; then removed the afterbirth completely, washed out the uterus with 1 to 5000 hot bichloride solution of mercury, and with exception of a rise of temperature that evening to 100.5, which I regarded as due to shock and not to sepsis, and gave her a hypodermic of a twelfth of a grain of heroin, she had a good night's sleep and made an uninterrupted recovery. Both mother and child are in good condition.

In all the cases I was ably assisted by Drs. Sappington and Kleberg.

---

The diagnosis of tuberculosis and cancer will make better progress when family history is utterly ignored.—*American Journal of Surgery.*

---

Chronic Arthritis.—Dr. Bayard Holmes, in *Lancet-Clinic*, says: "The treatment of a chronic arthritis which has been demonstrated to be non-tuberculous consists in at least three distinct maneuvers. The first is designed to increase the elimination, promote nutrition and do away with the anemia; the second is the eradication of the primary infection upon which the arthritis is dependent; and the third is the repair of the joint, the removal of the deformity and the restoration of the joint and the surrounding muscles to their normal condition."

